# Interactogeneous: Disease Gene Prioritization Using Heterogeneous Networks and Full Topology Scores

**DOI:** 10.1371/journal.pone.0049634

**Published:** 2012-11-19

**Authors:** Joana P. Gonçalves, Alexandre P. Francisco, Yves Moreau, Sara C. Madeira

**Affiliations:** 1 Knowledge Discovery and Bioinformatics Group, INESC-ID, Lisbon, Portugal; 2 Computer Science and Engineering Department, Instituto Superior Técnico, Technical University of Lisbon, Lisbon, Portugal; 3 Electrical Engineering Department, Katholieke Universiteit Leuven, Leuven, Belgium; University of Turin, Italy

## Abstract

Disease gene prioritization aims to suggest potential implications of genes in disease susceptibility. Often accomplished in a guilt-by-association scheme, promising candidates are sorted according to their relatedness to known disease genes. Network-based methods have been successfully exploiting this concept by capturing the interaction of genes or proteins into a score. Nonetheless, most current approaches yield at least some of the following limitations: (1) networks comprise only curated physical interactions leading to poor genome coverage and density, and bias toward a particular source; (2) scores focus on adjacencies (direct links) or the most direct paths (shortest paths) within a constrained neighborhood around the disease genes, ignoring potentially informative indirect paths; (3) global clustering is widely applied to partition the network in an unsupervised manner, attributing little importance to prior knowledge; (4) confidence weights and their contribution to edge differentiation and ranking reliability are often disregarded. We hypothesize that network-based prioritization related to local clustering on graphs and considering full topology of weighted gene association networks integrating heterogeneous sources should overcome the above challenges. We term such a strategy Interactogeneous. We conducted cross-validation tests to assess the impact of network sources, alternative path inclusion and confidence weights on the prioritization of putative genes for 29 diseases. Heat diffusion ranking proved the best prioritization method overall, increasing the gap to neighborhood and shortest paths scores mostly on single source networks. Heterogeneous associations consistently delivered superior performance over single source data across the majority of methods. Results on the contribution of confidence weights were inconclusive. Finally, the best Interactogeneous strategy, heat diffusion ranking and associations from the STRING database, was used to prioritize genes for Parkinson’s disease. This method effectively recovered known genes and uncovered interesting candidates which could be linked to pathogenic mechanisms of the disease.

## Introduction

Biomarkers play a crucial role in modern medicine as a means to improve accuracy in diagnosis, prognosis and treatment. In recent years, large-scale studies on the involvement of genes in disease have been empowered both by advances in high-throughput techniques and the proliferation of accessible resources of biological data. Manually inspecting the outstanding amounts of available ‘omics’ data is infeasible and can only be achieved by narrowing down the search space, often through a pre-selection of genes yielding higher potential using automatic recognition methods. Disease gene prioritization addresses this problem by generating ordered lists of candidates pertaining a particular gene-disease relatedness score. Most computational strategies follow a guilt-by-association approach, where new genes are implicated based on a measure of relatedness with a set of other genes known to be involved in the occurrence of a particular phenotype. Relatedness can be defined based on a number of properties, including intrinsic and functional [1]. Similarly, some approaches exploit the topology of biological networks and capture protein interactions, regulatory links or metabolic pathways into the score. Within the network-based scheme, candidates are sorted based on connectivity with the known genes, under the assumption that genes contained in a particular network substructure, exhibiting dense linkage, or sharing pathways tend to participate together in biological processes. Additional effort has been put into complementing the network analysis with information extracted from expression data [2] or genome-wide association studies [3]. Existing relations with phenotypes and among phenotypes themselves have also been used to aid in the generation of new hypothesis (yet undiscovered associations between genes and diseases) [4]–[6].

Many studies [2]–[12] have relied on interaction networks to unravel novel disease genes. Concerned about the quality of data, most authors have focused on interactions obtained from ‘trusted’ sources. While curated physical interactions are generally preferred in this regard [4]–[6], [8], [10], [12], they often lead to insufficient coverage of the human genome. In this context, the integration of complementary knowledge from heterogeneous sources is essential to achieve an understanding of the system as a whole and obtain well populated networks. This assumption has motivated the design of methods based on multiple sources of biological data, including co-expression, pathway, function, and literature. However, many studies devise their own integration from a limited number of datasets, which is likely to narrow the sources and network size [3], [7], [9], [12]. A reasonable alternative would be to analyze the extensive information provided by heterogeneous networks generated by successful efforts on larger scale assembly [11], [13], [14]. Despite the increased risk of false positives, these networks are denser, less biased toward a particular evidence, and therefore likely to be more robust to noise. Finding the tradeoff between the pros and cons of heterogeneity is however a challenge.

A key feature in network prioritization with known genes is the set of paths considered when calculating disease-relatedness. Popular approaches use neighborhood measures based on direct links between the candidates and the known genes [8], [11]. A major drawback in this setting is to assume that every pair of functionally related genes pertains a direct link. This is likely to impose a bias toward established information, hampering the exploitation of new hypotheses. Some methods overcome the issue by focusing on reachable genes, namely via shortest paths [7], [15]. Nonetheless, this measure lacks resolution, as the longest shortest path connecting a gene pair is typically short (small world networks). It also ignores redundant paths with different configurations, reportedly characteristic of biological networks [16], and indicative of interaction strength or robustness. Full topology strategies incorporate all alternative paths between candidates and disease genes into the score [3], [5], [6], [9], [10], [17]. This is expected to compensate for missing linkage and ultimately mitigate sparsity and small world effects, while promoting a comprehensive understanding of the system. In this context, can full topology scores consistently outperform path-restrictive measures in prioritization?

Random walk or diffusion kernel methods arise as natural choices to devise full topology scores and their application to gene prioritization has been proven effective [3], [5], [6], [9], [10], [12], [17]. Basically, they propagate a signal expressing the prior knowledge on the disease through the network and accumulate a gene-disease score for every gene, later used to sort the candidates. If the network is represented as a graph, this problem relates to retrieving the nodes most ‘similar’ to a group of interest and is known as local clustering. The major advantage of local clustering is the ability to direct the search toward the relevant areas of the graph based on the prior knowledge. In contrast, global clustering partitions the whole network in an unsupervised manner and thus tends to be more appropriate for problems in which candidates are to be distributed across multiple clusters, such as protein function prediction [18], [19]. Notably, local methods have been shown to deliver more functionally coherent clusters when prior knowledge is available [20]. Additionally, they proved superior to unsupervised or semi-supervised global partitioning also in the context of disease gene prioritization using physical protein-protein interactions [12]. Diffusion methods are often believed to be computationally expensive. However, this is not necessarily the case and depends mostly on the approximation in use. Iterative versions of these methods are time efficient, accommodating well for oversized networks [17]. Typical practice also involves performing a large number of iterations, independently of the actual number of steps needed to obtain a reasonable solution or the steady-state probability. According to the literature, limited diffusion should be sufficient for ranking purposes, as most nodes will be reached in a few steps (small world effect) [21], [22]. Whether focusing on a limited neighborhood or considering full topology, network-based approaches should be able to discern the relative importance of interactions in order to improve score reliability. Incorporating confidence weights may be a solution for this, but it is sometimes ignored [9], [10], [12].

In this work, we advocate for a particular category of network-based prioritization strategies, which we term Interactogeneous. These combine full topology scores computed using local clustering on graphs or diffusion kernels over confidence weighted gene association networks integrating evidence from heterogeneous sources. We formulate the hypothesis that Interactogeneous strategies should outperform approaches missing at least one of the described features. We investigate the validity of this statement in three case studies concerning the prioritization of genes for 29 diseases: (1) gene-disease score - full topology *vs* neighborhood and shortest paths; (2) network - heterogeneous associations *vs* single source interactions; (3) confidence weights and other parameters. Finally, we test the effectiveness of the best prioritization strategy to recover prior knowledge and suggest genes likely to play a role in Parkinson’s disease (PD).

## Methods

In this section, we first outline formal concepts and the prioritization problem. We then describe the prioritization methods and networks included in this study. An association or interaction network can be described as a weighted undirected graph 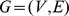, where 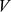 is the set of vertices and 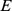 is the set of edges. Each vertex in *V* and edge in *E* correspond to a gene and an association between two genes, respectively. Let *A* and *D* denote the adjacency and diagonal matrices of *G*, respectively. 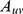 is the weight 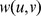 of edge 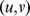 between source 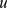 and target 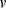. Let also 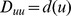, where 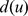 is the sum of the weights of the edges for which 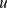 is the source, or 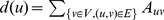. Disease gene prioritization is here formulated as obtaining a ranking on *V* given a set 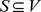 of seeds corresponding to known disease-related genes.

### Gene-disease Prioritization Score

**Figure 1:**
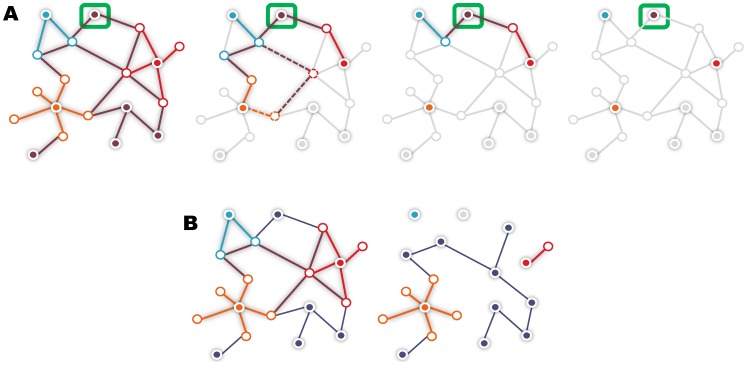
Impact of score and network properties on the prioritization. Subfigure A) shows the impact of the prioritization score properties. Subfigure B) shows the impact of network properties. Disease genes are depicted in red, light blue and orange. Edges between a disease gene and its neighbors have the same color as the disease gene node. Subfigure A) pictures the paths considered when computing the score for a given candidate (green box) using full topology, shortest paths, direct neighbor overlap (EndNet), and direct neighbor approaches, respectively. Edges in grey are ignored. Full topology is the most comprehensive strategy, as it considers all paths. In shortest paths, the alternative (shortest) path between the candidate and the orange gene (dashed line) is not taken into account. Contribution of additional paths of different lengths is also ignored. Using the direct neighbor overlap, the candidate can still be linked to the disease, but with no contribution from the orange gene. With direct neighbors, the candidate receives a score of 0. Subfigure B) shows the effect of dense *vs* sparse interactions and good *vs* poor genome coverage. Heterogeneous associations (left) *vs* single source interactions (right), the latter with absent genes (blue gene) and lack of connectivity (red gene).

One of our goals is to evaluate whether a full topology measure is more informative than a neighborhood or shortest paths score (Figure 1). In this section, we introduce the specific prioritization methods analyzed in this study. Presented computational complexities assume that the graph is implemented as a collection of adjacency lists.

#### Full topology

Full topology scores were obtained with PageRank [23] or heat diffusion [21] algorithms, often used to assess the relevance of Web pages based on linkage. Although related in formulation and expected to generate similar results under appropriate parameterization, heat diffusion has been shown to outperform PageRank [21], which in turn has been shown equivalent to HITS [24] and K-Step Markov [25] methods in disease gene prioritization [10]. Recently, Navlakha and Kingsford [12] showed that the random walk method in the work of Köhler et al. [9], precisely PageRank, was also superior to approaches based on direct neighbors, unsupervised and semi-supervised graph partitioning, and an additional method based on network flow [5]. Both heat diffusion and PageRank propagate a signal expressing the relevance of the genes in the context of the disease through the network to compute a gene-disease score for every gene in the network. Relevances are represented in the form of a preference vector, *p*, containing as many entries as genes in the network. Distinct schemes can be used to initialize *p*, whose instantiation is denoted by 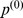. The most common is to attribute preference 1 or 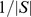 to entries corresponding to genes in the seed set (known disease genes), and 0 to the remaining entries, 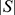 being the size of the seed set. Gene-disease scores are computed iteratively in *N* steps and take 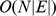 time.

*Heat diffusion* is a discrete approximation of the heat kernel [26] first introduced by [21], in which the rate of diffusion is controlled by a non-negative parameter *t*, the diffusion coefficient. The iterative score update for gene *v* is given by
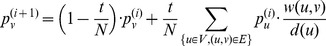
(1)


*PageRank with priors* is an extension of the original PageRank to consider a custom initial distribution of the scores [23], [25]. A parameter 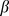, or “back probability”, denotes the probability of jumping to an initial node at each step. In this context, 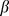 is also a factor that indicates the persistence of the initial preference for such node. The iterative equation is given by
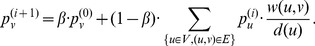
(2)


#### Neighborhood

Three neighborhood scores were calculated. *Disease neighbor weighting* [11] sums, for each gene *v*, the weight of every edge linking *v* to a disease gene. *Disease neighbor counting* [8] counts, for each gene *v*, the number of direct neighbors known to be involved in the disease. Computing any of these two scores for all genes takes 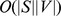. *Disease neighborhood overlap* [27] defines the score of gene *v* as the relative overlap between two sets containing: (i) disease genes and their direct neighbors; and (ii) *v* and its direct neighbors. This is the score computed by the Endeavour tool [27] for network data and is 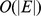 with the first set stored as a hashtable. We refer to it as EndNet.

#### Shortest paths

Different versions of shortest paths can be defined [7], [15]. In this work, we consider the score of gene *v* to be the average of the lengths of the shortest paths between *v* and the disease genes. Computation of the shortest paths scores for all genes takes 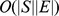 using breadth-first search [28].

### Network

Absent genes and missing links affect the neighborhood of genes, and thus the overall network topology on which network-based methods rely to compute gene-disease scores, thus hampering the discovery of novel genes (Figure 1). Essentially, we assessed two groups of networks: one containing gene associations integrated from multiple sources of biological evidence, named heterogeneous (reasonable coverage, denser); the other holding gene associations derived from a specific type of data such as physical interactions, here referred to as single source (poor coverage, sparser).

#### Heterogeneous associations

The *STRING* database [14] gathers information from conserved genomic neighborhood, gene fusion events and phylogenetic co-occurrence, co-expression, interaction and pathway databases, literature and large-scale experiments. It relies on a Bayesian framework to integrate established knowledge with putative associations returned by prediction algorithms or transferred from model organisms. We downloaded STRINGv8.2 (2009-10-18 to 2010-05-26, EnsEMBL r46), containing evidence from MINT [29], HPRD [30], BIND [31], DIP [32], BioGRID [33], KEGG [34], Reactome [35], IntAct [36], PID [37] and GO complexes [38]. We converted the original STRING protein association network into a gene association network. Protein names were mapped to their encoding genes using own parsing of EnsEMBL files [39]. Specifically, we addressed the case of genes encoding multiple proteins as follows. For each pair of interacting genes, we took the edge of maximum (integrated) weight linking any pair of proteins encoded by such genes.

*HEFalMp* is a network of functional gene associations supported by physical, genetic, sequence, or co-expression evidence [13], with genes identified by HGNC symbols [40]. Confidence scores are derived by naive Bayes classifiers trained on a gold standard of data from KEGG [34], HPRD [30], Reactome [35], PID [37], GO [38], Pfam [41] and the curated GSEA pathways [42]. Bayesian regularization with mutual information scores is used to penalize redundancy and prevent overconfident prediction. HEFalMp is a complete graph, where most interactions (≈80%) pertain scores below 0.16. We pruned less relevant edges based on a confidence score threshold, as those did not show to provide performance improvement. Experiments with subsets of HEFalMp obtained at different confidence cutoffs showed that the best results were achieved with 0.2 or lower (denser networks), decreasing for larger cutoff values (sparser networks). We used the pruned HEFalMp network (cutoff weight 0.2) in all the experiments described below. As it is clear from context, we may refer to it simply by HEFalMp throughout the text.

#### Single source associations

**Figure 2:**
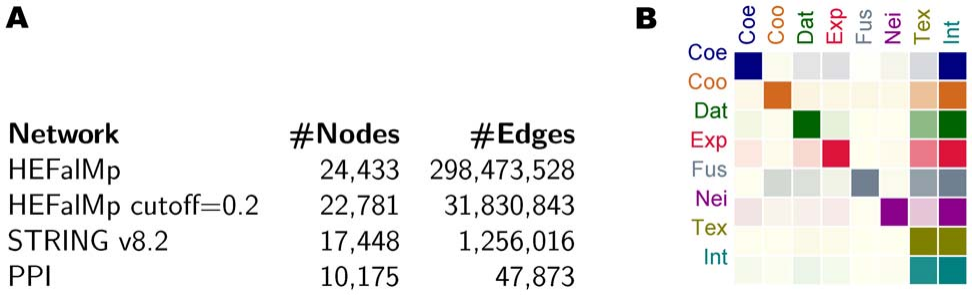
Network sizes and overlap between STRING sources. Subfigure A) shows the network sizes. Subfigure B) shows the overlap between STRING sources. In B), the color intensity of cell 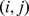 denotes the size of the intersection between the sets of edges in networks *i* (row) and *j* (column) relative to the size of *i*. Darker and lighter indicate larger and smaller. From left to right: co-expression, co-occurrence, database, experimental, fusion, neighborhood, text mining and integrated.

We parsed a network of physical protein-protein interactions (PPI) from the NCBI Entrez Gene repository [43], using Entrez Gene names. It yielded data from BIND [31], BioGRID [33] and HPRD [30]. We also generated independent networks, one per type of evidence in STRINGv8.2 (Figure 2), and used the protein to gene network conversion procedure (see previous subsection: Heterogeneous associations). We adopt a similar terminology to that used by the authors in the original STRING publication for each of the sources, as follows. ST-Co-expression denotes associations derived from similarity of expression profiles between the pairs of genes across different expression datasets. ST-Co-occurrence corresponds to associations expressing phylogenetic co-occurrence, that is, co-occurrence of the pairs of genes across genomes. ST-Database corresponds to manually curated physical interactions. ST-Experimental refers to interactions detected in high-throughput experimental essays such as Y2H. ST-Fusion denotes associations between genes involved in gene fusion events. ST-Neighborhood comprises associations between genes exhibiting conserved genomic neighborhoods.

#### ST-Text mining and ST-Integrated

An additional network was extracted from STRING, ST-Text mining, containing associations based on the co-occurrence of gene pairs in literature abstracts. We prefer to classify ST-Text mining as a heterogeneous network, as it implicitly includes information from alternative sources (results presented in the literature are usually supported on experimental findings, among others). Upon separation of the distinct sources from STRING, it was necessary to convert the resulting protein-protein networks into gene-gene networks. However, choosing the edge of maximum weight between every pair of genes before or after the integration (as described for STRINGv8.2 above) would not yield identical integrated confidence weights. In order to preserve consistency between the interaction weights in the single source and integrated networks, eventually perturbed by the protein to gene conversion, we also built an integrated network from the single source networks, which we name ST-Integrated. We first performed the protein to gene network conversion per source, and then integrated all sources for every edge following the integration approach in the original STRING. Subfigure B shows the intersection between ST-Integrated and each of the extracted single source networks. Experiments with the ST-Integrated network were performed exclusively for comparison with the individual STRING sources. Throughout the text we refer to the original STRING network as STRINGv8.2 and to the custom integrated STRING network as ST-Integrated, whenever it matters. Additionally, we may use the term STRING to refer to either or both of these networks whenever they exhibit similar performances.

### Known Disease-related Genes

Prioritization strategies were evaluated using 620 known disease-related genes automatically selected from OMIM [44] as in [27], spanning 29 diseases with an average of 21 genes per set. Seeds were originally denoted by EnsEMBL [39] identifiers. They were respectively converted to Entrez Gene [43] and HGNC [40] symbols for the PPI network and HEFalMp networks based on own parsing of mappings retrieved from the corresponding Entrez Gene and HGNC FTP repositories.

### Evaluation Scheme

**Figure 3:**
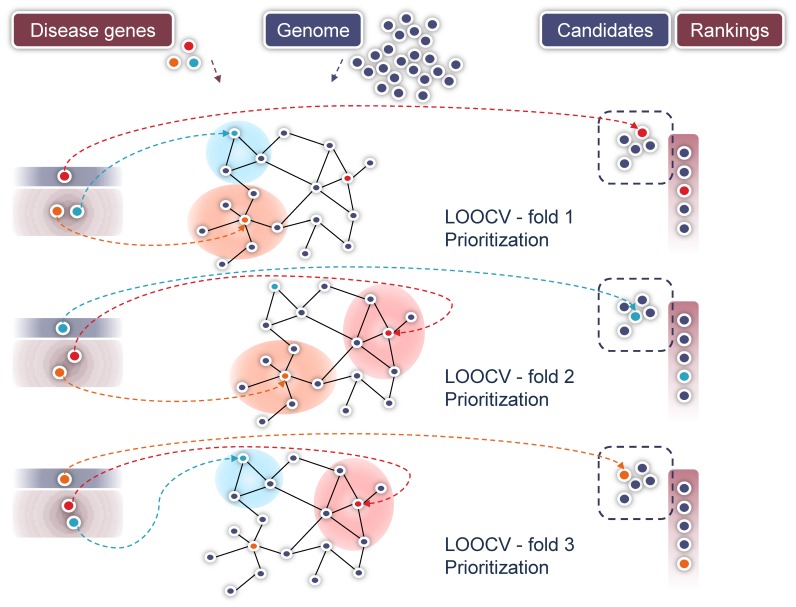
Evaluation scheme. In each leave-one-out cross-validation fold for a given disease, a different gene is retained from the set of known disease genes (red, blue, orange). The remaining genes known to be associated with that particular disease are mapped onto the network and used as prior knowledge (training set) to compute gene-disease scores for all the genes in the network. A test set, including the left-out gene and a set of candidates previously sampled from a pool of genes (the genes in a network or the intersection of the sets of genes in different networks), is sorted according to the obtained gene-disease scores. The performance is then determined by assessing the position of the left-out gene in the ranked test set. We average the overall and per disease results obtained in 10 complete leave-one-out cross-validation runs, each using a distinct set of candidate genes.

Leave-one-out cross-validation tests were conducted (Figure 3). For each disease and parameter setting, 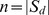 rankings were generated, where 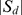 is the size of set 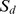 containing genes related to disease 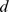. Each ranking was obtained by retaining a different gene from set 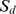 and using the remaining genes in the set as seeds in the prioritization task (training set). From the resulting (genome-wide) scoring, we retrieved an ordered list maintaining the relative positions of the left-out gene and a set of additional candidates previously selected from the network (test set). We repeated the cross-validation procedure using 10 distinct sets of candidates. These were previously generated by selecting genes uniformly at random, either from the set of genes in a network or the intersection of the sets of genes in several networks, when applicable, and excluding those genes in the seed sets. Ideally, the left-out gene should rank at the top. We assessed the overall and per disease performance averaged over the 10 distinct runs based on four measures, which assess the position of the left-out genes in the ranked test sets: counts of left-out genes in the top 10 and 20 ranks, area under the ROC curve, and mean average precision.

#### Area under the ROC curve

Prioritizers return a real-valued function used to define a strictly ordered ranking. Let positive and negative classes of a binary classification problem denote association and non-association with disease, respectively. A binary outcome can be produced by sweeping an imposed cutoff over the ranks and plotting the corresponding receiver operating characteristic curve (ROC) of the true positive rate as a function of the false positive rate. In this case, the left-out gene ranking higher than the cutoff implies its association with the disease. AUC denotes the area under ROC and fits in 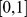, where a perfect prioritizer yields AUC 1 and random guessing achieves approximately 0.5. It estimates the probability that a randomly chosen positive example ranks higher than a negative one, being equivalent to the Wilcoxon-Mann-Whitney statistic. We computed AUC scores based on the procedure outlined by Fawcett [45].

#### Mean average precision

MAP denotes the mean of the average precision of the rankings obtained for all left-out genes. Average precision combines precision, the probability that a randomly selected gene associated by the prioritizer is in fact related, and recall, the probability that a randomly selected gene known to be involved in the disease is associated by the prioritizer. It averages the precisions computed by truncating the ranked list after each relevant item is found, where precision at rank *r* is the ratio between the number of relevant items and the total number of items retrieved at *r* [46]. In our setting, the only relevant item is the left-out gene and precision at *r* is thus 0 before it has been found, or 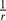 otherwise. MAP scores vary between values close to 0 for random guessing, and 1 for an ideal ranking, respectively.

## Results and Discussion

In this section, we investigate the impact of network features and ranking strategy on the prioritization task. We aim to validate three claims concerning the performance of Interactogeneous stategies against alternative approaches: (1) heterogeneous associations should outperform single source data, as multiple evidence increases coverage/density and reduces bias toward individual sources; (2) a full topology score resuming all paths is potentially more informative than measures based on direct links or shortest paths, deriving a whole-system perspective and avoiding excessive focus on well studied genes; (3) incorporating confidence weights should increase reliability, as it is expected to diminish the effect of false positives. Finally, we evaluate the effectiveness of the method to discover genes putatively associated with Parkinson’s disease (PD).

### Case Studies

Leave-one-out cross-validation tests were conducted. For each disease, in each evaluation run, a different gene was retained from the set of disease genes (training set). Prioritizers were applied to compute a score for every gene in the network, taking training genes as seeds. A test set was formed by adding the retained gene to a previously generated set of candidates. The genes in the test set were then ranked according to their gene-disease scores. We performed leave-one-out cross-validation in order to be able to evaluate all diseases, even those yielding only a few known genes, in most networks. Results of 5-fold cross-validation on the larger disease sets are also discussed below and made available in Supporting Information S1. Candidates for the test set were selected uniformly at random from the set of genes in the network (or the intersection of the sets of genes in a collection of networks, when applicable), excluding those genes in the seed sets. We preferred random selection of genes for the test set over collecting genes in the neighborhood of the known disease genes [5], [7], as in the latter case the genes tend to be disease genes as well, and our aim is to assess whether the method effectively discerns novel disease genes from the rest rather than whether a disease gene stands out among its peers. Given the sampling procedure involved in the selection of the remaining candidates for the test set, we generated 10 distinct sets of candidates and averaged the performances over the 10 complete cross-validation runs for the 29 diseases, each using a particular candidate set. We used a test set size of 100, which accounts for a good representation of the population: the genes in a network.

Full topology rankings were obtained with heat diffusion (HDiffusion) and PageRank (PRank). A measure based on shortest paths (SPaths) and three neighborhood scores, namely disease neighbor weighting (NWeight) or counting (NCount) and neighborhood overlap (EndNet), were selected for comparison. Heterogeneous association (STRING, including STRINGv8.2 and ST-Integrated versions; ST-Text mining; HEFalMp) and single source interaction (other STRING sources and NCBI PPI) networks were assessed (see Methods). Direct neighbor methods are highly dependant on the connectedness of the network. In fact, when using direct neighbor methods on single source networks, more than half of the genes yield score zero upon prioritization. The lack of resolution of direct neighbor and SPaths methods additionally affects the ranking in a negative way, whereby many genes pertain equal scores (commonly zero and non-zero values, respectively). In these cases, a strict order can no longer be defined based on the ranking scores. The solution adopted to mitigate these issues was to position the left-out gene in the median rank among those ranks occupied by all the genes yielding the same ranking score. For HDiffusion and PRank, seed scores were initialized to 1 and 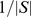, respectively, where 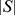 denotes the size of the seed set. Presented results use the best parameter setting. Performance is indicated by AUC, MAP, and percentages of left-out genes ranked in top 10 and top 20.

#### Gene-disease score: full topology *vs* neighborhood and shortest paths

**Figure 4:**
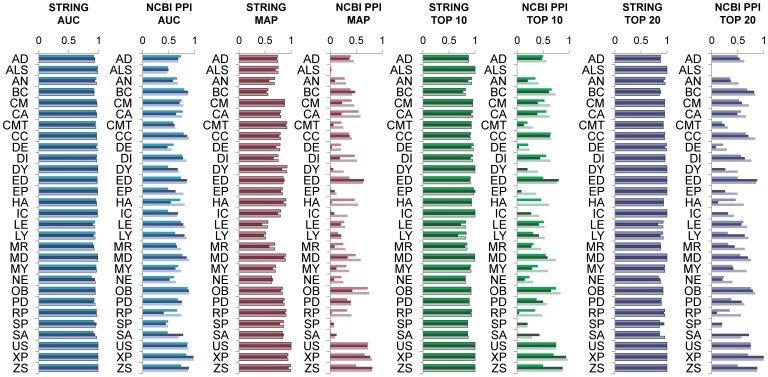
Prioritization results per disease. Each pair of charts shows the average of a different evaluation measure (left to right: AUC, MAP, top 10 and top 20) for 10 complete leave-one-out cross-validation runs (see Methods) on STRINGv8.2 (first chart) or the PPI network (second chart). A distinct, previously generated, candidate set was used in each of the 10 runs. For each disease the top, middle and bottom bars denote the results of NWeight, SPaths and HDiffusion. The list of disease acronyms, from top to bottom: AD, Alzheimer’s disease; ALS, amyotrophic lateral sclerosis; AN, anemia; BC, breast cancer; CM, cardiomyopathy; CA, cataract; CMT, Charcot-Marie-Tooth disease; CC, colorectal cancer; DE, deafness; DI, diabetes; DY, dystonia; ED, Ehlers-Danlos syndrome; EP, epilepsy; HA, hemolytic anemia; IC, ichthyosis; LE, leukemia; LY, lymphoma; MR, mental retardation; MD, muscular dystrophy; MY, myopathy; NE, neuropathy; OB, obesity; PD, Parkinson’s disease; RP, retinis pigmentosa; SP, spastic paraplegia; SA, spinocerebellar ataxia; US, Usher syndrome; XP, xeroderma pigmentosum; ZS, Zellweger syndrome.

HDiffusion was the best method overall, consistently performing on top in all networks (Tables 1, 2 and 3, and Figure 4). PRank performed well on the heterogeneous networks, although it did not achieve outstanding scores compared with its competitors. Notably, it delivered robust performance across the different networks and exhibited good results on the NCBI PPI network. While SPaths seemed to position itself in second place (together with NCount) when considering the heterogeneous networks, STRINGv8.2 and HEFalMp, it proved more sensitive to the absence of knowledge in the single source NCBI PPI network, where its performance dropped significantly. The two neighborhood methods NWeight and NCount achieved reasonably good performance using heterogeneous associations. NWeight was particularly impressive and basically head-to-head in terms of evaluation scores with HDiffusion on the heterogeneous networks, STRINGv8.2 and HEFalMp (Tables 1 and 2). Nevertheless, NWeight and NCount failed to recover the knowledge contained in the NCBI PPI network (Table 3). In such network, only 44.3% of the 526 seed genes present in the NCBI PPI network (or 84.8% of the total of 620 originally in the seed sets), were attributed a score larger than zero and thus deemed to have some relation with the corresponding disease by NWeight or NCount. The fact that most of those left-out genes achieved the top 10 and nearly all were placed in the top 20 highlights the binary decision nature of these neighborhood methods (Tables 1, 2 and 3), whereby left-out genes either rank at the very top or are otherwise missed. This behavior stems both from the methods themselves and the way in which the evaluation was conducted. On one hand, neighborhood measures attribute scores only to those genes which are direct neighbors of the disease genes. On the other hand, all candidates except the left-out gene are randomly selected from the network. Although the association of the genes with a particular disease will depend on the connectedness of the disease genes in each particular network, the direct neighbors are likely to represent in general a limited proportion of the genes in the network. As a result, the probability of the majority of the selected candidates yielding score 0 is large and the left-out gene will likely stand out among the remaining candidates whenever it is a neighbor of some disease gene(s). EndNet was the method with poorest results in both heterogeneous networks. Surprisingly it showed up immediately after HDiffusion in the performance table for the NCBI PPI network (Tables 1, 2 and 3). EndNet still missed nearly a third of the disease associations that could potentially be recovered from the NCBI PPI network, attributing a score larger than zero to 72.1% of the 526 seed genes in the NCBI PPI network (84.8% of the total number of seeds). EndNet also exhibited a binary effect, although less pronounced than that of NWeight and NCount, as 48.9% and 60.6% of the genes in the network were placed respectively in the top 10 and top 20. The relative performances observed overall were maintained when considering the results per disease (Figure 4), where the differences between the methods were again more noticeable in the results for the single source network and HDiffusion leaded the performance rankings. When using the NCBI PPI network, neighborhood methods NWeight and NCount could not rank any of the genes associated with dystonia, epilepsy, ichtyosis, spastic paraplegia, or spinocerebellar ataxia, in the top 20. Likewise, all the genes known to be related to deafness, hemolytic anemia and retinis pigmentosa missed the top 20 when ranking with SPaths. Overall, the results corroborate the reasoning that full topology methods can compensate for missing links by exploiting higher order neighborhoods and path redundancies, proving particularly relevant when analyzing single source networks. They also provide higher resolution scores, thus more informative and robust rankings.

**Table 1:** Leave-one-out cross-validation results of all methods on STRING.

Method	AUC	MAP	TOP 10	TOP 20	SRec	DRec	SEval
**HDiffusion**	**0.957±0.004**	**0.731±0.023**	**90.3±0.9**	**94.4±0.9**	**100**	**100**	**100**
PRank	0.926±0.013	0.516±0.098	79.4±5.6	89.5±2.6	100	100	100
EndNet	0.848±0.014	0.242±0.049	53.4±2.8	73.3±3.7	100	100	100
**NWeight**	**0.950±0.003**	**0.733±0.022**	**90.3±0.9**	**94.2±0.8**	**96.8**	**100**	**100**
NCount	0.944±0.004	0.679±0.027	87.8±1.6	93.3±0.8	96.8	100	100
SPaths	0.943±0.005	0.673±0.027	86.2±1.6	91.7±0.9	100	100	100

Results of each tested prioritization method on the STRINGv8.2 network. Mean and standard deviation of four evaluation measures (AUC, MAP, and percentage of left-out genes ranked in tops 10 and 20), obtained for 10 complete leave-one-out cross-validations on the 29 disease sets using 10 distinct previously generated candidate sets. ‘SRec’: percentage of left-out genes (from the total number of seeds in the original seed sets: 620) effectively ranked, that is, yielding a ranking score larger than zero. ‘DRec’: percentage of recovered diseases among the 29 diseases with seeds (a disease is recovered if at least one of its left-out genes obtained a ranking score larger than zero). ‘SEval’: percentage of left-out genes (from the total number of seeds originally in the seed sets: 620) in the network. All evaluation measures, AUC, MAP, TOP 10 and TOP 20, were computed taking into account only the left-out genes present in each network (SEval), rather than all the genes originally in the seed sets. Parameters: HDiffusion (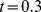, 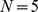), PRank (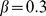, 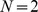).

**Table 2:** Leave-one-out cross-validation results of all methods on HEFalMp.

Method	AUC	MAP	TOP 10	TOP 20	SRec	DRec	SEval
**HDiffusion**	**0.829±0.020**	**0.252±0.032**	**48.1±5.4**	**68.5±4.9**	**99.2**	**100**	**99.2**
PRank	0.767±0.027	0.138±0.030	33.4±6.0	53.8±5.8	99.2	100	99.2
EndNet	0.743±0.035	0.115±0.030	27.3±6.2	47.9±8.3	99.2	100	99.2
**NWeight**	**0.827±0.019**	**0.261±0.030**	**49.2±5.6**	**68.8±4.6**	**96.1**	**96.6**	**99.2**
NCount	0.782±0.028	0.147±0.022	37.5±6.5	59.3±5.9	96.1	96.6	99.2
SPaths	0.783±0.028	0.147±0.022	37.5±6.5	59.3±5.9	99.2	100	99.2

Results of each tested prioritization method on the HEFalMp network with edge weight cutoff 0.2. Mean and standard deviation of four evaluation measures (AUC, MAP, and percentage of left-out genes ranked in tops 10 and 20), obtained for 10 complete leave-one-out cross-validations on the 29 disease sets using 10 distinct previously generated candidate sets. ‘SRec’: percentage of left-out genes (from the total number of seeds in the original seed sets: 620) effectively ranked, that is, yielding a ranking score larger than zero. ‘DRec’: percentage of recovered diseases among the 29 diseases with seeds (a disease is recovered if at least one of its left-out genes obtained a ranking score larger than zero). ‘SEval’: percentage of left-out genes (from the total number of seeds originally in the seed sets: 620) in the network. All evaluation measures, AUC, MAP, TOP 10 and TOP 20, were computed taking into account only the left-out genes present in each network (SEval), rather than all the genes originally in the seed sets. Parameters: HDiffusion (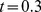, 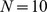), PRank (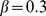, 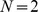).

**Table 3:** Leave-one-out cross-validation results of all methods on the NCBI PPI network.

Method	AUC	MAP	TOP 10	TOP 20	SRec	DRec	SEval
**HDiffusion**	**0.771±0.012**	**0.375±0.031**	**53.9±1.7**	**63.7±2.2**	**82.9**	**100**	**84.8**
PRank	0.738±0.018	0.197±0.050	44.2±5.1	57.4±2.3	82.9	100	84.8
EndNet	0.748±0.010	0.290±0.031	48.9±2.4	60.6±1.3	61.1	100	84.8
NWeight	0.695±0.004	0.317±0.027	42.4±1.4	44.2±0.2	37.6	100	84.8
NCount	0.694±0.003	0.294±0.023	42.1±2.2	44.3±0.0	37.6	100	84.8
SPaths	0.701±0.018	0.214±0.024	36.6±2.5	49.4±2.6	82.9	100	84.8

Results of each tested prioritization method on the NCBI PPI network. Mean and standard deviation of four evaluation measures (AUC, MAP, and percentage of left-out genes ranked in tops 10 and 20), obtained for 10 complete leave-one-out cross-validations on the 29 disease sets using 10 distinct previously generated candidate sets. ‘SRec’: percentage of left-out genes (from the total number of seeds in the original seed sets: 620) effectively ranked, that is, yielding a ranking score larger than zero. ‘DRec’: percentage of recovered diseases among the 29 diseases with seeds (a disease is recovered if at least one of its left-out genes obtained a ranking score larger than zero). ‘SEval’: percentage of left-out genes (from the total number of seeds originally in the seed sets: 620) in the network. All evaluation measures, AUC, MAP, TOP 10 and TOP 20, were computed taking into account only the left-out genes present in each network (SEval), rather than all the genes originally in the seed sets. Parameters: HDiffusion (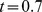, 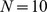), PRank (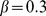, 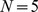).

#### Network: heterogeneous *vs* single source

Heterogeneous associations in STRING outperformed both HEFalMp and the NCBI PPI network using every method tested in this study and every of the 10 candidate sets generated from each pool of genes (Tables 4 and 5). Considering the results of HDiffusion, the best method overall in our study, STRING evaluated considerably higher than curated protein interactions (STRINGv8.2, ST-Integrated or ST-Text mining against ST-Database or NCBI PPI network in Tables 3 and 4). Approximately 83–84% of all the genes in the disease seed sets ranked in the top 10 on STRING, opposed to 49–55% of 85% of the original seeds on the NCBI PPI network (42–47%) and 54–55% of the 59% of the original seeds on ST-Database (≈32%). ST-Experimental performed comparably to slightly better than the physical interaction sources, NCBI PPI and ST-Database. Additionally, STRING proved more informative than HEFalMp, delivering superior performance in every evaluation measure (Tables 4 and 5). No relevant knowledge could be retrieved from ST-Co-occurrence, and poor performance was achieved using ST-Co-expression or ST-Neighborhood, the sparsest networks. Text mining data excelled. Not surprisingly, as literature contributes to 88% of the interactions, whereas the remaining 12% rely exclusively on other sources (Figure 2). It is worth to note in this regard that literature generally reports information latent in (and conclusions derived from) alternative sources, resulting in a comprehensive network with heterogeneous information. HEFalMp performed comparably to and worse than the larger single source networks (NCBI PPI, ST-Database, and ST-Experimental), considering the results obtained using candidates from the smaller and larger pools, respectively, and excluding the known disease genes absent from each network in its evaluation (Tables 4 and 5). Using HEFalMp, only 33–51% of the 99% of the genes found in the network ranked in the top 10 (33–50%). Additionally, it outperformed the three smaller single source networks (ST-Co-expression, ST-Co-occurrence and ST-Neighborhood) using both pools of candidates. The performance of HEFalMp indicates that, although density and coverage tend to be important properties, they do not present a guarantee of outstanding rankings, to which topology will certainly contribute. Network construction features, including the selection of sources and integration scheme, as well as the ranking approach and its network search strategy and score definition should also play relevant roles in this regard. In this study, none of the prioritization methods seemed to be able to translate the topology and weighting scheme of HEFalMp into a more effective disease candidate gene ranking. Overall, we noticed a tendency for heterogeneous networks (STRINGv8.2, together with ST-Integrated and ST-Text mining, and HEFalMp) to deliver superior performance relative to single sources (Tables 4 and 5). Although we excluded absent genes from the evaluation measures, it is also important to note that using heterogeneous networks we could assess 99% to 100% of the known disease genes, while single sources missed 11% to 92% of those seeds.

**Table 4:** Leave-one-out cross-validation results of HDiffusion on ten networks.

Network	AUC	MAP	T10	T20	SRec	DRec	SEval
NCBI PPI network	0.765	0.354	55%	64%	83%	100%	85%
HEFalMp cutoff = 0.2	0.771	0.274	51%	63%	99%	100%	99%
ST-Co-expression	0.665	0.269	37%	44%	13%	52%	13%
ST-Co-occurrence	0.490	0.164	21%	24%	9%	69%	18%
ST-Database	0.753	0.376	55%	65%	58%	93%	59%
ST-Experimental	0.752	0.365	55%	64%	89%	100%	89%
ST-Neighborhood	0.622	0.176	32%	39%	6%	34%	8%
**ST-Text mining**	**0.926**	**0.683**	**84%**	**90%**	**100%**	**100%**	**100%**
**ST-Integrated**	**0.918**	**0.665**	**84%**	**89%**	**100%**	**100%**	**100%**
**STRING v8.2**	**0.920**	**0.665**	**84%**	**90%**	**100%**	**100%**	**100%**

Leave-one-out cross-validation results of HDiffusion on the PPI, HEFalMp cutoff = 0.2, STRING sources, and integrated STRING networks. Scores averaged over 10 distinct sets of candidates previously sampled from the pool of 277 genes contained in the intersection of the sets of genes in all the networks except ST-Fusion (excluding the seeds). Parameters 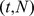 from top to bottom networks: 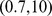, 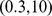, 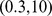, 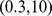, 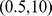, 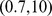, 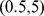, 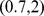, 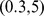, 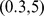. ‘SRec’: percentage of left-out genes (from the total number of seeds in the original seed sets: 620) effectively ranked, that is, yielding a ranking score larger than zero. ‘DRec’: percentage of recovered diseases among the 29 diseases with seeds (a disease is recovered if at least one of its left-out genes obtained a ranking score larger than zero). ‘SEval’: percentage of left-out genes (from the total number of seeds originally in the seed sets: 620) in the network. All evaluation measures, AUC, MAP, TOP 10 and TOP 20, were computed taking into account only the left-out genes present in each network (SEval).

**Table 5:** Leave-one-out cross-validation results of HDiffusion on the larger networks.

Network	AUC	MAP	T10	T20	SRec	DRec	SEval
NCBI PPI network	0.726	0.316	49%	58%	83%	100%	85%
HEFalMp cutoff = 0.2	0.696	0.171	33%	47%	99%	100%	99%
ST-Database	0.749	0.353	54%	63%	58%	93%	59%
ST-Experimental	0.749	0.345	53%	61%	89%	100%	89%
**ST-Text mining**	**0.923**	**0.657**	**84%**	**89%**	**100%**	**100%**	**100%**
**ST-Integrated**	**0.919**	**0.644**	**83%**	**89%**	**100%**	**100%**	**100%**
**STRING v8.2**	**0.920**	**0.642**	**83%**	**89%**	**100%**	**100%**	**100%**

Leave-one-out cross-validation results of HDiffusion on the PPI, HEFalMp cutoff = 0.2, the larger STRING sources, and integrated STRING networks. Scores averaged over 10 distinct sets of candidates previously sampled from the pool of 4092 genes contained in the intersection of the sets of genes in the largest networks (and excluding the seeds). ‘SRec’: percentage of left-out genes (from the total number of seeds in the original seed sets: 620) effectively ranked, that is, yielding a ranking score larger than zero. ‘DRec’: percentage of recovered diseases among the 29 diseases with seeds (a disease is recovered if at least one of its left-out genes obtained a ranking score larger than zero). ‘SEval’: percentage of left-out genes (from the total number of seeds originally in the seed sets: 620) in the network. All evaluation measures, AUC, MAP, TOP 10 and TOP 20, were computed taking into account only the left-out genes present in each network (SEval).

#### Confidence weights and other parameters

In general, methods incorporating edge weights outperformed those ignoring them, in the networks where weights were available, namely STRING and HEFalMp (Tables 1, 2 and 3). We further investigated the contribution of confidence weights to the final scores by comparing the previous results on STRING and HEFalMp (using the original weights) with the results obtained after setting the weights of all interactions to 1. SPaths, EndNet and NCount methods maintained their performances as expected, since they ignore edge weights. Using the original edge weights on STRING promoted an addition of approximately 9 genes (1.5% of the total number of genes) ranked in the top 10 with HDiffusion. NWeight lead to around 16 additional genes in the top 10 (2.5%) and PRank to 24 genes more (3.9%). Original weights on HEFalMp increased the number of genes in the top 10 by approximately 46 genes (7.5%) with HDiffusion, and 58 genes (11.7%) with NWeight, compared to the non weighted version of HEFalMp. PRank was not able to take advantage of the weights on HEFalMp. Notably, HDiffusion was able to increase its performance in 13.4% (STRING) and 12.6% (HEFalMp) of the margin that was still available for improvement by incorporating edge weights. For NWeight, the increase represented 20.5% and 18.7% of the margin for improvement on STRING and HEFalMp, respectively. Concerning edge differentiation, the relative improvement potentiated by the use of differentiated weights is equivalent in both networks, HEFalMp and STRING. Additionally, NWeight revealed more sensitive to these weights than HDiffusion. From these results, we observe that the contribution of weights is likely to translate into fine adjustments to the computed ranking scores rather than substantial changes in performance. In the case of a fully connected network (complete graph), such as HEFalMp, weights should be key to effectively differentiate the edges. Typically, a threshold is applied to discard edges of low confidence, contributing to the definition of a distinct network topology. In this context, edge weights are therefore supporting the consistently higher performances obtained on STRING relative to the remaining networks in this study. Edge weights thus influence network density and topology. Density is an important property and denser networks (up to an optimal point) are more likely to perform better. However, density is not an assurance of outstanding results, essentially because an increased number of edges does not necessarily imply an increase in the number of ‘true’ edges. Moreover, there is no guarantee that the weights of the edges in the network effectively reflect the ‘true’ order of importance between edges. In particular, a given edge could yield weight zero due to either its irrelevance or absence of information. Ultimately, this is influenced by the accuracy of the weights which also determines the network topology. Both full topology methods (HDiffusion and PRank) achieved maximum performance using a limited number of iterations, *N*, which was shown to be smaller for STRING and HEFalMp than for the PPI network. This could be explained by a stronger small world effect on the denser networks caused by the existence of shorter paths linking the pairs of nodes. Regarding the propagation rate, values 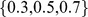 were tried for *t* and 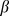. For different *t* and equal *N*, HDiffusion pertained variations in the orders of 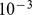 (AUC) and 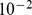 (MAP) on STRING and the PPI. PRank exhibited score changes for different 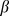 with equal *N* in the orders of 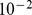 (AUC) and 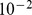 to 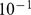 (MAP) on STRING and 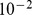 to 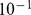 (AUC, MAP) on the PPI network. Overall, PRank was more sensitive to variations in this parameter.

#### Leave-one-out *vs* k-fold cross-validation

Leave-one-out cross-validation is not usually the preferred approach for performance evaluation, as it can lead to optimistic results. Nevertheless, its choice is widely accepted in the literature in cases where the data is scarce. In this work, the size of the seed sets was reasonably small and would prevent the application of a standard 10-fold cross-validation. Similarly, a 5-fold approach would immediately exclude 4 networks and 19 diseases due to insufficient coverage of the seed sets by the networks. On the other hand, our study focused on relative rather than absolute performances, and the leave-one-out procedure is not expected to favor *per se* a specific method or class of methods, or a particular network or class of networks, in detriment of others. In this context, we aimed primarily at promoting a fair comparative evaluation procedure. For completeness, we conducted 5-fold cross-validation tests on the larger disease sets, those with 20 or more known disease genes present in the three major networks (STRING, HEFalMp and the NCBI PPI network). Such results are presented in Supporting Information S1. We observed that most performance measures remained relatively stable, which allowed to maintain the relative performances of the several methods and the various networks.

### Unveiling Genes Associated with Parkinson’s Disease

Parkinson’s disease (PD; OMIM #168600) is the second most common neurodegenerative disorder, after Alzheimer’s [47], [48]. Clinically, it is characterized by resting tremor, muscular rigidity, bradykinesia, and postural instability. Pathological evidence includes degeneration of midbrain dopaminergic neurons and formation of abnormal ubiquinated protein aggregates, Lewy bodies, and filamental structures, Lewy neurites [47]. Eighteen genetic loci (PARK1-18) have been related to PD [48]–[50]. Several genes have been reported to carry mutations affecting susceptibility to familial and idiopathic PD. Multifactorial genetic and environmental conditions are under study. Here, we assess the ability of prioritization to identify promising candidates for PD based on linkage with known genes.

#### Prior knowledge for Parkinson’s disease

The list of known PD related genes excludes recent findings and purposely lacks some of the well known disease causing factors in an attempt to assess the ability of the method to unravel novel and recover established knowledge on the disease. Additional noise was introduced with the inclusion of *protein kinase, AMP-activated, gamma 2 non-catalytic subunit* (*PRKAG2*), a gene involved in Wolf-Parkinson-White syndrome responsible for heart defects. All remaining genes have been linked to PD before. Mutations in genes *synuclein, alpha (non A4 component of amyloid precursor)* (*SNCA*) located in PARK1 and PARK4 and *ubiquitin carboxyl-terminal esterase L1 (ubiquitin thiolesterase)* (*UCHL1*) in PARK5 were identified in autosomal dominant families, while mutated *parkinson protein 2, E3 ubiquitin protein ligase (parkin)* (*PARK2*) in PARK2 and *parkinson disease (autosomal recessive, early onset) 7* (*PARK7*) in PARK7 have been implicated in autosomal recessive forms of monogenic PD [48], [51]. Four susceptibility genes not related with the PARK loci were also included. *Nuclear receptor subfamily 4, group A, member 2* (*NR4A2*), required for the development of dopaminergic neurons and associated with both autosomal dominant and sporadic PD [48]. Also, *microtubule-associated protein tau* (*MAPT*), for which several haplotypes were indicated to influence age at onset and risk of PD [48]. *NADH dehydrogenase (ubiquinone) flavoprotein 2, 24 kDa* (*NDUFV2*), involved in electron transfer from NADH to the mitochondrial respiratory chain where the electron acceptor is believed to be *ubiquinone* [52]. Finally, *cytochrome P450, family 2, subfamily D, polypeptide 6* (*CYP2D6*), encoding an enzyme involved in dopamine metabolism, was included due to its multiple polymorphisms affecting susceptibility to PD [53].

#### Involvement of prioritized genes in Parkinson’s disease

**Figure 5:**
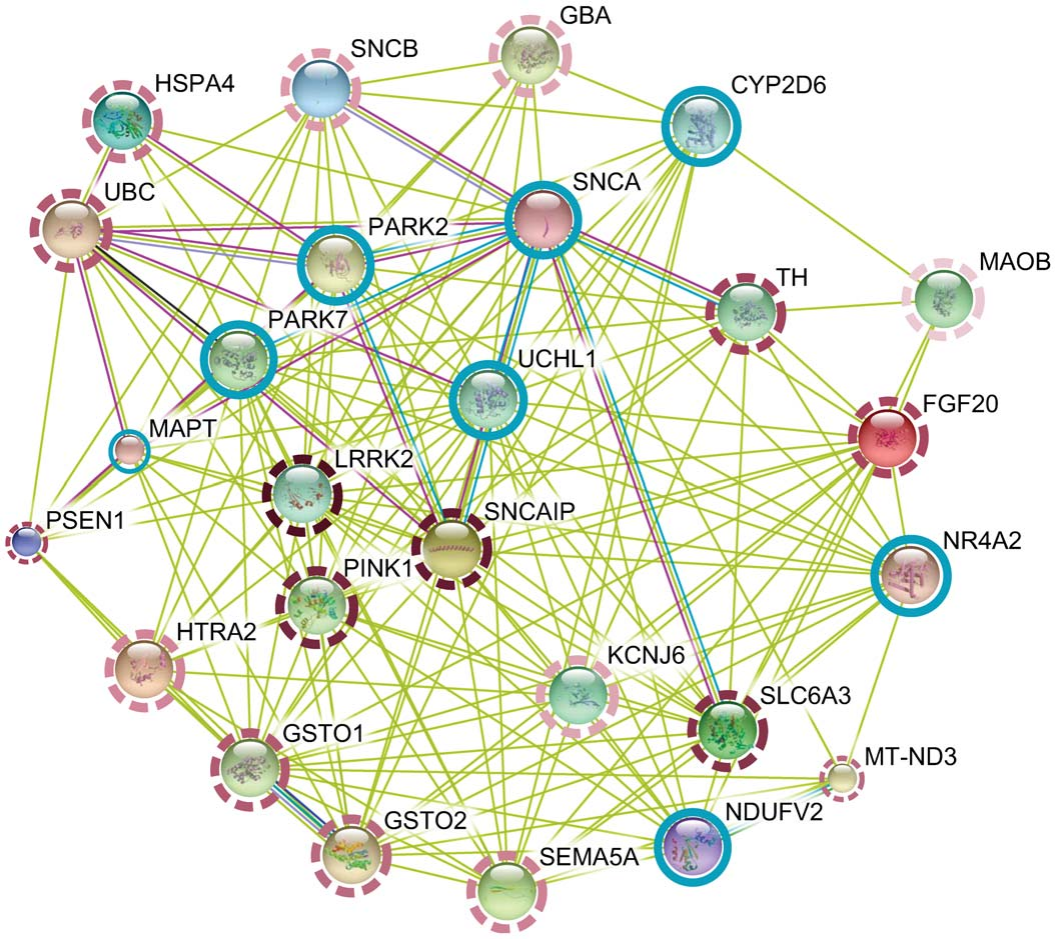
Prioritization results for Parkinson’s disease. Rings indicate whether genes are seeds (blue, full line) or top ranked genes (bordeaux, dashed line). Color intensity (dashed) varies with prioritization score: darker and lighter mean higher and lower in the ranking. Multiple association evidence is depicted in distinct colors. Only associations with confidence above 0.4 are shown. Figure obtained with STRING tools and adapted to express the ranking.

Heat diffusion was applied to STRING using the Parkinson seeds. *PRKAG2* ranked last among the seeds, confirming non-relatedness to PD. All seeds were filtered from the results. We investigate the first 20 genes, of which 18 could be associated with mechanisms of PD (Figure 5). We also refer the number of entries in PubMed where each gene name and PD words co-occur (between braces below), as a quantitative indicator of the knowledge underlying the literature.

In the top three, the *PTEN induced putative kinase 1* (*PINK1*, 269 entries) gene in PARK6 known to cause autosomal recessive early-onset PD and the *leucine-rich repeat kinase 2* (*LRRK2*, 488 entries) gene in PARK8 often related to autosomal dominant late-onset variant [48], [49], [51], followed by the *synuclein, alpha interacting protein* (*SNCAIP*, 62 entries) known to cooperate with *SNCA* and *PARK2* in the formation of Lewy bodies [54]. Interestingly, the *fibroblast growth factor 20* gene (*FGF20*, 12 entries) possessing broad mitogenic and cell survival activities, ranked in the top 10. Variation in *FGF20* has been linked to risk for PD, not confirmed by later studies [49]. Among the top 10 were also found *ubiquitin C* (*UBC*, 67 entries) and *glutathione S-transferase omega 1* and *2* (*GSTO1* and *GSTO2*, 7 entries each), hinted to influence age at onset in PD [55], followed by the *heat shock 70 kDa protein 4* (*HSPA4*, 74 entries). These genes are involved in mitochondrial activity, particularly linked to ubiquitination and chaperone function, whose dysfunction and impairment in PD leads to the degradation of proteins and accumulation of Lewy bodies [56]. Mitochondrial *HtrA serine peptidase 2* (*HTRA2*, 20 entries), located in PARK13 and found among the best 20, promotes cell death by binding apoptosis inhibitory proteins. Genetic variability in *HTRA2* has been reported to cause PD, though studies showing no association have also been published [48], [49], [51]. Neurotransmitters and ion channel receptors involved in idiopathic PD such as the sodium- and chloride-dependent neurotransmitter *solute carrier family 6 (neurotransmitter transporter, dopamine), member 3* (*SLC6A3*, 90 entries) and *potassium inwardly-rectifying channel, subfamily J, member 6* (*KCNJ6*, 8 entries) [56] ranked in tops 10 and 20, respectively. Enzymes involved in dopamine metabolism and biosynthesis, *tyrosine hydroxylase* (*TH*, 994 entries) [57], [58] and *monoamine oxidase B* (*MAOB*, 221 entries) [59], [60], ranked among best 10 and 20, respectively. Recent findings showed that heterozygous mutations in the *glucosidase, beta, acid* gene (*GBA*, 43 entries), top 20, significantly alter risk for sporadic and familial PD [49], [61]. Several genes pending confirmation ranked in the top 20. *Mitochondrially encoded NADH dehydrogenase 3* (*MT-ND3*, 1 entry) is a core subunit of the mitochondrial membrane respiratory chain NADH dehydrogenase complex I. Although *MT-ND3* has not been specifically linked to PD, dysfunction of complex I is considered the major mitochondrial defect in PD [62] and mutations in other subunits (e.g. *ND2*, *ND5*) have been filed. *Presenilin 1* (*PSEN1*, 21 entries) is known to carry mutations causing Alzheimer’s disease (AD). Impairment of presenilin-mediated signaling pathways leading to presynaptic dysfunction has been suggested as a converging event before neurodegeneration in AD and PD [63]. Contradictory evidence exists on the *sema domain, seven thrombospondin repeats (type 1 and type 1-like), transmembrane domain and short cytoplasmic domain, (semaphorin) 5A* (*SEMA5A*, 5 entries) gene, involved in axonal guidance during neural development, connectivity maintenance and repair [64]. Similarly, *synuclein, beta* (*SNCB*, 43 entries), homologous to *SNCA*, has been found highly expressed in the substantia nigra [65] and referred as a potential inhibitor of *SNCA* aggregation and fibrillization [66]. *SNCB* might also influence age at onset [67].

Overall the ranking reflected the contribution of additional sources as, in several cases, genes with scarce literature evidence ranked higher than others with widely spread reference (*FGF20* and *KCNJ6* against *TH* and *MAOB*, for instance).

### Conclusion

In this work, we sought for a robust network-based disease gene prioritization strategy able to address some limitations of current methods. We advocated a preference for what we termed Interactogeneous strategies, a synonym for full topology scores based on weighted gene associations derived from multiple sources, and claimed that these should exhibit superior performance relative to existing approaches missing at least one of such features.

The impact of network configuration, score definition and parameters was analyzed in studies involving the prioritization of genes for 29 diseases. In our study, full topology scores outperformed neighborhood and shortest paths measures given their consistent top results across the different types of networks. The gap between the two kinds of methods was inexistent when using heterogeneous networks, but rather particularly noticeable when single source networks (including the popular manually curated networks of physical protein-protein interactions) were considered. These results support the reasoning that incorporating full topology with higher order neighborhoods and alternative paths (redundancies) potentiates a more comprehensive understanding of the interactome and ability to compensate for missing linkage. They further corroborate the findings in previous studies giving the lead to full topology scores against other measures based on direct neighbors, shortest paths and graph partitioning on protein-protein interaction networks [5], [9], [12]. Additionally, our results suggest that the superiority should not be generalized to every network. Nevertheless, the ability to retrieve knowledge from an incomplete network, from which a large part of the information might be missing, is most appealing to the research community. Our study confirmed that local clustering is suitable for an effective identification of the information relevant to the disease when prior knowledge is available, as previously reported in the literature [12], [20]. In addition, heterogeneous associations from the STRING database revealed advantageous when compared to the networks of its individual sources, a curated physical protein-protein interaction source and also an alternative functional network, HEFalMp. Integration of knowledge retrieved from complementary biological data proved crucial to achieve a more comprehensive and informative understanding of the system, given its ability to generate networks less biased toward a particular evidence and less prone to suffer the negative effect of false positives. Our results further suggest that the impact of confidence weights on the ranking scores is mainly produced by discerning relevant from irrelevant associations leading to the definition of the network topology, rather than providing accurate relationships of importance between the different associations. Nonetheless, methods performed better when incorporating edge weights in networks where they were available. We also observed that density is not likely to present an important property by itself. In network-based prioritization, it is more important to ensure that the relevant edges in the network cover most of the ‘true’ associations between genes, and obviously hold as little of the false associations as possible. Ultimately, the selection of data sources, integration scheme, score definition and network search strategy are all likely to influence the quality of the rankings output by network-based methods.

Interactogeneous strategies proved successful. We used the best performing technique, combining heat diffusion ranking with the STRING network, to prioritize genes potentially involved in Parkinson’s disease. Known susceptibility genes widely referred in literature or databases were recovered. Analysis of the top ranked genes further revealed a number of genes with scarce or controversial evidence, most of which could be related to mechanisms of Parkinson’s disease: (1) impairment of mitochondrial activity and dopamine metabolism with oxidative stress leading to apoptotic death of dopaminergic neurons; (2) disruption of the ubiquitin proteasome system and consequent protein degradation with formation of Lewy bodies; (3) presynaptic dysfunction caused by perturbation of developmental signaling pathways; (4) compensatory mechanisms for cell survival and repair upon stress.

## Supporting Information

Supporting Information S1 **5-fold cross-validation results.** This document contains the results obtained for all methods (HDiffusion, PRank, EndNet, NWeight, NCount, SPaths) on three networks (STRINGv8.2, HEFalMp and NCBI PPI network) using the larger disease sets in a 5-fold cross-validation evaluation scheme.
(PDF)
