# Supplementary material for: Interactogeneous: Disease Gene Prioritization Using Heterogeneous Networks and Full Topology Scores
Source: PLoS One. 2012 Nov 19;7(11):e49634. doi: 10.1371/journal.pone.0049634 (PMC3501465; doi:10.1371/journal.pone.0049634)
Supplement: Supporting Information S1 — 5-fold cross-validation results. This document contains the results obtained for all methods (HDiffusion, PRank, EndNet, NWeight, NCount, SPaths) on three networks (STRINGv8.2, HEFalMp and NCBI PPI network) using the larger disease sets in a 5-fold cross-validation evaluation scheme. (PDF) [file pone.0049634.s001.pdf]

Interactogeneous - supporting information

Joana P. Gonçalves[1,2,*], Alexandre P. Francisco[1,2], Yves Moreau[3], Sara C. Madeira[1,2,*]
1 Knowledge Discovery and Bioinformatics Group, INESC-ID, Lisbon, Portugal
2 Computer Science and Engineering Department, Instituto Superior Técnico, Technical University of Lisbon, Lisbon, Portugal
3 Electrical Engineering Department, Katholieke Universiteit Leuven, Leuven, Belgium
∗ E-mail: jpg@kdbio.inesc-id.pt, sara.madeira@ist.utl.pt

Abstract

This document contains additional results and supplementary information on the evaluation procedure.

1 Evaluation procedure

In the manuscript, we present the results obtained using a leave-one-out cross-validation (LOOCV) procedure. This approach is widely used by the machine learning community for performance assessment of prediction methods and model selection [1, 2]. It is known to provide an unbiased estimator for the true expected prediction error, but also to lead to large variance in some cases [1]. Note that the variance mentioned in the literature would refer, in this case, to the fluctuations observed across the rankings obtained for the several LOOCV folds of a given disease. This follows from the fact that each LOOCV performance estimate is obtained by testing on a single data point, while in other standard k-fold cross-validation approaches (10-fold and 5-fold, for instance) each estimate is essentially an average of the results obtained for several data points. The standard deviations presented in our manuscript denote the variation between the overall scores obtained for the 29 diseases over 10 complete LOOCV runs, each using a distinct set of candidates generated prior to the experiments, in which the only element that varies is the set of candidates (the additional genes added to the left-out gene in the test set). This follows from the fact that all prioritization methods evaluated in the manuscript are deterministic. The LOOCV procedure is also deterministic, given that each and every gene is tested on its own in a different LOOCV run, and all remaining genes are used for training.

1.1 Results of 5-fold cross-validation

As the results of LOOCV tend to be optimistic, LOOCV is not usually the first choice for performance evaluation. However, its use is widely accepted in the literature in cases where the data is scarce. In the case of our study, performing 10-fold cross-validation was infeasible and 5-fold cross-validation would imply setting aside 19 diseases and 4 networks. In addition, there is no particular reason why LOOCV should favor a particular method or network. Given that our manuscript focuses on comparisons between methods/networks rather than on absolute performances, LOOCV should provide as fair comparison as any other form of cross-validation. For completeness, we herein present the results obtained using 5-fold cross-validation on the 10 diseases yielding 20 or more known disease genes in each of the three major networks: STRINGv8.2, HEFalMp and the PPI network from NCBI (Table 1). For evaluation purposes, whenever several of the left-out genes in a given 5-fold ended up included in a group of genes with the same ranking score, the left-out genes were positioned around the median rank for that group (given that at most one hit per rank is assumed). We observed that the relative performances of the different methods and networks were maintained. The measures exhibit an overall slight decrease as it would be expected, in contrast with the optimism of LOOCV.

Table 1. 5-fold cross-validation results of all methods on (a) STRINGv8.2, (b) HEFalMp and (c) the NCBI PPI network. Scores were averaged over 10 complete cross-validation runs for the 29 diseases using distinct sets of candidates previously generated by drawing genes uniformly at random from the network. All evaluation measures, AUC, MAP, TOP 10 and TOP 20, were computed taking into account only the left-out genes present in each network (SEval), rather than all the genes originally in the seed sets (620).

(a) STRINGv8.2. HDiffusion ($t = 0.3$, $N = 5$), PRank ($\beta = 0.3$, $N = 2$).

Method	AUC	MAP	TOP 10	TOP 20
**HDiffusion**	**0.954** ± **0.003**	**0.831** ± **0.005**	**80.2** ± **1.0**	**90.7** ± **0.7**
PRank	0.924 ± 0.001	0.694 ± 0.003	69.9 ± 0.3	84.1 ± 0.4
EndNet	0.842 ± 0.001	0.398 ± 0.002	44.0 ± 0.4	66.9 ± 0.3
**NWeight**	**0.948** ± **0.004**	**0.830** ± **0.006**	**80.5** ± **1.0**	**90.7** ± **0.7**
NCount	0.942 ± 0.003	0.799 ± 0.004	77.3 ± 0.6	89.5 ± 0.6
SPaths	0.942 ± 0.002	0.791 ± 0.004	76.6 ± 0.6	88.5 ± 0.6

(b) HEFalMp. HDiffusion ($t = 0.3$, $N = 10$), PRank ($\beta = 0.3$, $N = 2$).

Method	AUC	MAP	TOP 10	TOP 20
**HDiffusion**	**0.825** ± **0.003**	**0.380** ± **0.005**	**41.1** ± **1.0**	**64.3** ± **0.9**
PRank	0.792 ± 0.001	0.340 ± 0.001	34.8 ± 0.3	53.6 ± 0.2
EndNet	0.750 ± 0.001	0.231 ± 0.001	24.8 ± 0.3	46.0 ± 0.2
**NWeight**	**0.827** ± **0.003**	**0.390** ± **0.006**	**42.3** ± **1.1**	**65.4** ± **0.8**
NCount	0.781 ± 0.003	0.271 ± 0.006	32.2 ± 0.6	55.7 ± 0.9
SPaths	0.782 ± 0.003	0.271 ± 0.006	32.2 ± 0.6	55.7 ± 0.9

(c) NCBI PPI network. HDiffusion ($t = 0.7$, $N = 10$), PRank ($\beta = 0.3$, $N = 5$).

Method	AUC	MAP	TOP 10	TOP 20
**HDiffusion**	**0.774** ± **0.007**	**0.469** ± **0.009**	**50.1** ± **1.0**	**62.5** ± **0.9**
PRank	0.762 ± 0.004	0.393 ± 0.005	46.8 ± 0.6	60.8 ± 0.7
EndNet	0.753 ± 0.007	0.397 ± 0.010	46.0 ± 1.2	59.1 ± 1.1
NWeight	0.684 ± 0.007	0.383 ± 0.012	39.5 ± 1.4	41.8 ± 1.4
NCount	0.684 ± 0.007	0.378 ± 0.011	39.8 ± 1.5	42.1 ± 1.4
SPaths	0.697 ± 0.003	0.310 ± 0.005	34.1 ± 0.9	48.2 ± 0.7

References

1. Hastie T, Tibshirani R, Friedman J (2009) The Elements of Statistical Learning. Springer.

2. Arlot S, Celisse A (2010) A survey of cross-validation procedures for model selection. Statistics Surveys 4: 40–79.
